# Fecal Incontinence: Prevalence, Severity, and Quality of Life Data from an Outpatient Gastroenterology Practice

**DOI:** 10.1155/2012/947694

**Published:** 2011-09-25

**Authors:** Eva H. Alsheik, Thomas Coyne, Sara K. Hawes, Laleh Merikhi, Scott P. Naples, Nandhakumar Kanagarajan, James C. Reynolds, Scott E. Myers, Asyia S. Ahmad

**Affiliations:** ^1^Division of Gastroenterology and Hepatology, Drexel University College of Medicine, 219 North Broad Street, 5th Floor, Philadelphia, PA 19107, USA; ^2^Cabarrus Gastroenterology Associates, Concord, NC, USA; ^3^Department of Medicine, Drexel University College of Medicine, Philadelphia, PA 19102, USA

## Abstract

*Background*. The prevalence of fecal incontinence varies tremendously as a result of inadequate data collection methods. Few office-based studies have assessed the prevalence of fecal incontinence and none have looked at modifiable risk factors or effect on quality of life. *Design, Settings, Patients, and Main Outcome Measures*. Five hundred patients who visited our inner city, university-based gastroenterology practice, were asked about symptoms of fecal incontinence. We also retrospectively reviewed 500 charts to identify the frequency of patient-physician reporting of fecal incontinence. *Results*. Of the 500 patients that were directly questioned, 58 (12%, 43 women, 15 men) admitted to fecal incontinence compared to 12 (2.4%) in the retrospective arm. Patients with fecal incontinence and loose/watery stool reported the lowest quality of life scores. While the average severity score was similar between men and women, women had a significantly lower average quality of life score (3.04 versus 2.51; *P* < 0.03). *Conclusions*. The identification of fecal incontinence increases when patients are directly questioned. Identifying and treating patients with loose stool is a potential strategy to improve quality of life in this patient population. In men and women with similar severity of fecal incontinence, women have a significantly lower quality of life.

## 1. Introduction

Fecal incontinence (FI) is defined as the inadvertent passage of stool, soiling, or excessive escape of flatus. The prevalence of FI varies among studies because of differing definitions of this disorder, patients' reluctance to report symptoms, and inadequate data collection methods [1]. In the general community, the prevalence ranges from 0.4% to 18% [2–12]. Office-based studies document that 13% to 29% of patients in primary care and specialty clinics admit to FI when asked [7, 10, 11, 13, 14]. Studies from obstetrics and gynecology, urogynecology, and antenatal outpatient clinics report prevalences between 5.6% and 29% [9, 14, 15]. Johansen and Lafferty are the only group who has sampled patients from primary care and gastroenterology outpatient offices. However, this study was limited to a predominantly Caucasian population and did not evaluate the effect of FI on quality of life (QOL) [16].

The difference in prevalence of FI between men and women has similarly yielded variable results. The discrepancies are largely due to inconsistent data collection methods, variable ages, and reluctance to report symptoms. A large-scale systematic review found that 0.8% of men and 1.6% of women aged 15 to 60 years reported FI. In those older than 60 years, the prevalence increased to 5.1% in men and 6.2% in women. The most recent epidemiologic survey cites a similar prevalence in men and women (8.9% versus 7.7%), whereas other studies cite a higher prevalence in men (20% versus 11%, *P* < 0.015) [6, 17]. None, however, has examined the gender-specific effect on symptom severity and QOL.

It is evident from the aforementioned studies that the prevalence rises when FI is directly addressed. If incontinence does not pertain to the patient's chief complaint, however, many physicians may not inquire. Unfortunately, this perpetuates the “do not ask, do not tell” cycle.

Better attempts to identify and treat patients with FI are essential, especially among physicians such as internists, gynecologists, and gastroenterologists, who are the most likely to treat this devastating condition.

We hypothesized that we could increase the identification of patients with FI by direct questioning during a routine gastroenterology office visit. We also hypothesized that we could determine if the severity of FI symptoms would correlate with quality of life. Our study intended to investigate these two hypotheses by adding the symptom of FI to our standardized gastrointestinal review of symptoms. We intended to evaluate QOL and severity of FI symptoms by administering validated questionnaires. In addition, we aimed to identify modifiable risk factors within this population that could be addressed and treated early to prevent the need for invasive procedures.

## 2. Methods

The study was conducted at a Drexel University College of Medicine outpatient office. This office is an inner-city, single-specialty university-based practice comprised of 8 gastroenterologists and 6 gastroenterology fellows who see outpatients with all types of gastrointestinal and liver disorders. Two of the 8 gastroenterologists specialize in motility disorders. The Drexel University College of Medicine Institutional Review Board approved this study.

The patients from this practice reflect an inner-city tertiary care population. More than half of our patients are African American and have an annual income of $25,000 or less. Approximately, a third of our patients have attended college or higher educational degrees. An equal number of men and women attend our practice. New patients are routinely asked to fill out a detailed but general review of systems while they wait to see our physician. During the visit, our history and physical forms (both new and follow-up) prompt our physicians to directly discuss a focused gastroenterology review of systems. Our gastrointestinal review of systems includes nine upper GI symptoms (dysphagia, odynophagia, heartburn, dyspepsia, abdominal pain, nausea, vomiting, melena, and weight loss) and eight lower GI symptoms (diarrhea, constipation, changes in bowel movements, decreased stool caliber, tenesmus, urgency, bleeding, and pain). FI is not included in either review-of-systems intake form. For this study, FI was added to the focused gastrointestinal review-of-systems intake form. Prior to the start of the study, all gastroenterologists were notified about the addition of FI to the standard review of systems intake form and were reminded to ask patients about this symptom. No restrictions were placed on the language physicians used to discuss this symptom with patients, with some gastroenterologists referring to “fecal incontinence” while others using terms such as “leakage or soiling.”

During our study period, 500 consecutive patients (both new and returning) visiting our gastroenterology practice were asked about FI during the gastrointestinal review of systems. Patients who said yes were asked to enroll in the study and underwent informed consent procedures. Physicians then verbally administered three questionnaires to the enrolled patients. The first questionnaire included demographic factors; known medical, surgical, and obstetric risk factors; medications; duration of symptoms; need and frequency of pad or diaper use; whether FI had ever been addressed by their health care providers (The Appendix). The second questionnaire consisted of the fecal incontinence severity index (FISI), which we used to assess the frequency and type of stool loss. The third questionnaire consisted of the fecal incontinence-specific American Society of Colon and Rectal Surgeons quality of life questionnaire (FIQL), which evaluates the impact of FI on coping, embarrassment, depression, and lifestyle [18]. We chose to use these scales because they allow subjects to weigh their answers. In turn, an external weighing scheme is employed for analysis. In addition, the validated FISI and FIQL questionnaires are easy to use, concise, reliable, and validated [2, 19].

Our study also included a retrospective arm aimed to identify the number of patients who reported FI before it was included in the standardized gastrointestinal review of systems. In this part of the study, any documentation of fecal incontinence would be the result of independent questioning by the physician or voluntary admission by the patient. Every third chart from our file room was selected until we reached 500 charts. We excluded patients if they had been seen in our practice within 1 month of the start date of the study. The selected charts were then completely reviewed for documentation of FI during any visit. Information regarding severity and effect on QOL was not assessed in this group because of the retrospective nature of the review.

### 2.1. Statistical Analysis

The data obtained was analyzed statistically using Pearson's *χ* ^2^ test along with 95% confidence intervals (CIs) to compare groups of interest (men, women, and combined). Logistic regression analysis was used to identify independent associations with gender variables. A *P* value of <0.05 was considered statistically significant. We performed additional analyses using Microsoft Excel (Redmond, WA) *t* tests for comparison of subgroup variables.

## 3. Results

Five-hundred individuals were approached over 3 consecutive months in the prospective arm of the study. Fifty-eight (11.6%) reported symptoms of FI, and all of these patients agreed to participate in the study. Of the patients who reported FI, 74% were women (43) and 26% were men (15) with an average age of 51.7 years (range 22–84 years) and an average age of onset of 48.6 years (range 17–80 years). Approximately, 90% of the patients who reported FI had a high school education or higher. Seventy-two percent of patients with FI had either medical or surgical risk factors for FI. Patient characteristics, duration and frequency of incontinence are summarized in Table 1.

Only 2 of the 58 patients (3%) in the prospective arm presented to the office with a chief complaint of FI. The remaining 56 (97%) reported incontinence only on direct questioning.

**Figure 1:**
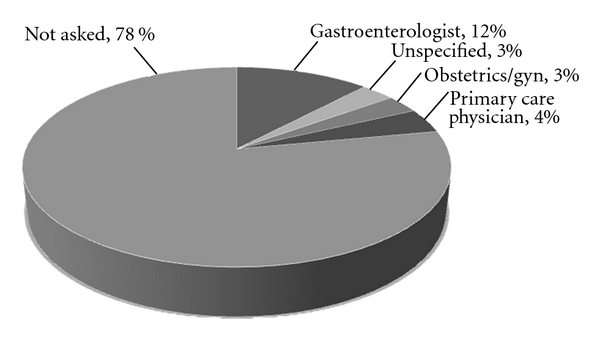
Percent of patients who had previously discussed with a physician, according to type of physician.

Thirteen of the 58 individuals (22%) had discussed their symptoms with a physician in the past (Figure 1). On retrospective chart review, only 12 (2.4%) of 500 patients had any mention of FI anywhere in their outpatient charts. Gastroenterologists that specialized in motility disorders were not more likely to ask about symptoms of FI in either the prospective or retrospective arms of the study. 

**Figure 2:**
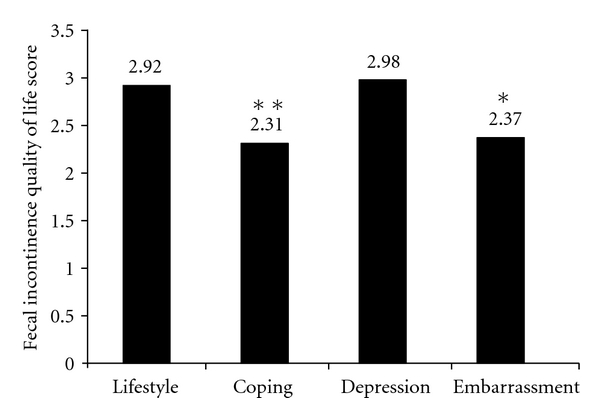
FIQL scores. *Significantly lower than lifestyle and depression scales (*P* < 0.006); **Significantly lower than lifestyle and depression scales (*P* < 0.003).

Twenty-eight of the 58 patients with FI (48%) reported a poor quality of life (FIQL score <2.5). Each of the four FIQL scales was independently scored. Patients with FI had a significantly lower coping score than either lifestyle score (2.31 versus 2.92, *P* < 0.002) or depression score (2.31 versus 2.98, *P* < 0.003). Similarly, FI patients had a significantly lower embarrassment score than either lifestyle (2.37 versus 2.92, *P* < 0.006) or depression score (2.37 versus 2.98 *P* < 0.001) (Figure 2). When combined QOL scores were compared among groups, patients with loose/watery stools had significantly lower QOL scores when compared against the groups with formed stools (*P* = 0.005), alternating loose/formed stools (*P* = 0.05), and all groups combined (*P* = 0.006) (Table 2). 

Thirty-two of 58 patients (55%) had high severity scores (FISI score >25). When severity scores were compared among groups, patients with formed stool had relatively lower severity scores than all other stool-consistency groups alone and combined, although the findings were not significant. 

At the time of visit, 41 of the 58 patients with FI (71%) reported altered stool form (loose/watery, hard, or alternating consistency). Of FI patients with loose/watery stool, six of 23 (26%) were taking laxatives and eleven of 23 (48%) were receiving no medical therapy at all, and only seven of 23 FI patients (30%) were reported using antidiarrheal agents. See Table 2 for a summary of stool consistency, medication use, severity, and quality of life scores.

Severity and QOL scores were evaluated separately for women and men in our cohort. The average FI severity score was not significantly different between men and women. However, women had a significantly lower average quality of life score than men (3.04 versus 2.51; *P* < 0.03).

**Figure 3:**
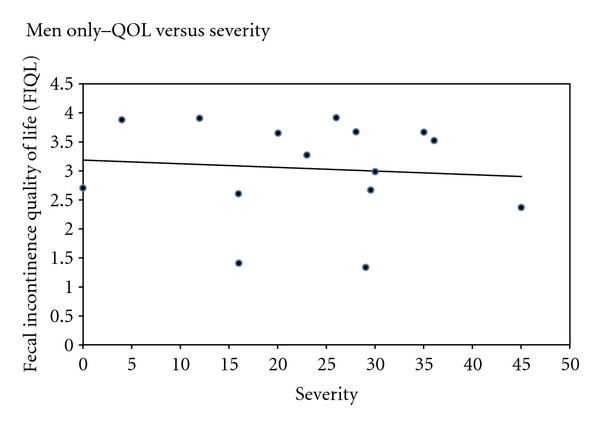
Quality of life as a function of severity of fecal incontinence in men (*P* = 0.75).

**Figure 4:**
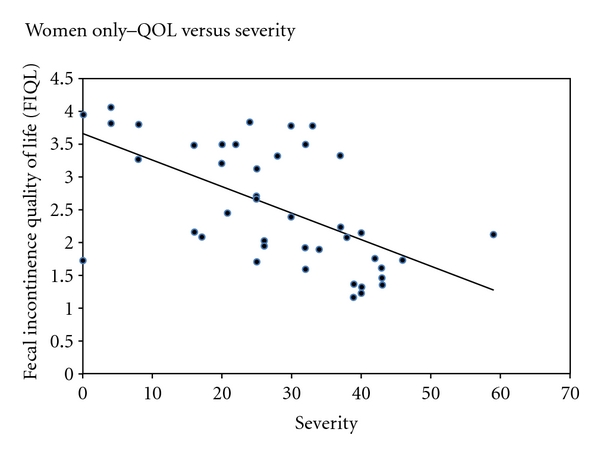
Quality of life as a function of severity of fecal incontinence in women (*P* < 0.001).

Pearson correlations were calculated for men, women, and men and women combined in regards to severity and QOL. For men, no significant correlation was found between severity and QOL (*r* = −0.09; *P* = 0.75; 95% CI −0.58 to 0.44). Conversely, a moderate correlation was found among women (*r* = −0.595; *P* < 0.001; 95% CI −0.68 to −0.36) (Figures 3 and 4). As the severity of FI increases, therefore, the QOL decreases in women. A moderate correlation between severity and quality of life was also found when men and women were analyzed together (*r* = −0.505; *P* < 0.001; 95% CI −0.68 to −0.28). 

## 4. Discussion

Although estimates of the overall prevalence of FI range from 0.4 to 18% in the general community, it is clear that the reported prevalence rises when patients are directly questioned about symptoms of FI. In this study, 12% of patients reported FI when directly asked, whereas a prevalence of only 2% was revealed in our retrospective arm. This latter finding is surprising given that we conducted the study in an academic practice with expertise in motility disorders. Furthermore, this is testament to the fact that if doctors do not ask, patients do not tell. Another unexpected finding is that almost 40% of patients that admitted to FI had a college education or higher. This is in contrast to our general patient population, in which two-thirds do not attain an education greater than high school. The cause of this unexpected finding was unclear. One possibility is that some of our less educated patients may have misunderstood the terminology that was used to describe FI. It is also plausible that certain groups of patients may be less willing to admit to FI due to the stigma that may be associated with it. Lastly, it is possible that more educated individuals have more bothersome work-related interruptions due to FI, which leads them to seek out medical attention. 

It was interesting that our FI patients had a reduced QOL predominantly as a result of issues with coping and embarrassment rather than depression and lifestyle issues. Although not officially validated, we ultimately chose to illustrate FIQL as a composite score of these main scales because we believe that the 4 scales of FIQL are simply facets of a total picture. In fact, Rockwood does state that with the FIQL “there is a sense that the overall quality of life is being assessed which is not true of other specialized scores such as those that assess depression or functional status.” [20] This approach should be further investigated.

While previously published studies examine the prevalence of FI among men and women or women alone, none focus on the gender-specific effect on QOL. Although no significant differences in severity scores were found between men and women, women with FI were found to have a significantly lower QOL. In addition, QOL significantly correlated with severity in women but not in men. This finding raises multiple questions. Are women more embarrassed by this issue than men, thereby more negatively affecting their quality of life? Conversely, are men more embarrassed by this issue and thus more reluctant to report their symptoms or admit their deteriorating QOL? Or are men less emotionally bothered by the soilage? Our small sample size limits our ability to make further generalizations and infer mechanistic differences between the genders. Larger studies on FI need to be conducted in order to uncover the relationship between gender and quality of life.

There are many risk factors for FI; however, only stool consistency is easily modifiable. That said, the best initial approach to fecal incontinence is to identify and target treatment for bowel consistency. Patients with formed stool reported lower severity scores than patients with loose, hard, or alternating bowel patterns. In addition, patients with loose stool had a significantly lower QOL than patients with alternating and formed stool consistencies. There are studies revealing that treating diarrhea-associated fecal incontinence with loperamide or fiber supplements is effective in the shortterm [21–24]. This is important considering that nearly 75% of patients who reported incontinence to loose/watery stool had not been taking appropriate anti-diarrheal therapy, and approximately 25% of patients were actually taking laxatives. It should be noted that patients with fecal impaction and resulting diarrhea and FI are generally treated with laxatives and enemas. Whether that occurred in a subset of our patients is unknown. Therefore, it is plausible that by simply inquiring about stool consistency, specifically diarrhea, and treating appropriately a physician may avoid the need for additional testing and referrals while simultaneously contributing to a patient's quality of life.

Our study had some potential limitations. For one, we did not administer an overall quality of life questionnaire to our patients in an attempt to limit the number of surveys that the patients had to complete. As a result, we are unable to compare the quality of life of our FI patients to other populations such as healthy individuals or those that suffered from urinary incontinence [20]. In addition, it could be considered a limitation that we did not use a strict, consistent definition for fecal incontinence when we approached our patients about this disorder. On the other hand, given the varied levels of education and communication skills of our patients, along with the sensitive nature of this issue, we felt that tailoring the individual interview instead of using a defined wording for FI was most appropriate. 

Therefore, asking patients directly about FI can lead to increased identification of this debilitating condition. This can be accomplished by prompting physicians to inquire about FI in a targeted review of gastrointestinal symptoms. This structured approach will likely yield a higher identification of FI patients. This will be especially true of physicians who tend to forget or consciously omit this symptom in their questioning. Furthermore, identifying and treating abnormal stool consistency in patients with FI is a potential strategy to reduce severity and improve quality of life. This intervention is especially important in women, who are more likely to be adversely affected by the severity of their symptoms. Patient and physician education should be stressed to shed light on this difficult and debilitating condition.

## Figures and Tables

**Table 1:** Patient Characteristics.

Patients, *n*	58
Female gender, *n* (%)	42 (74)
Patient age, years (range)	45 (22–84)
Onset age, years (range)	49 (17–80)

Education level, %	
Less than high school	9.3
High school	51.8
Attended college or greater	38.9

Patients with risk factors for FI, *n* (%)	72
Hemorrhoids	21 (36.0)
Hysterectomy^a^	12 (29.0)
Irritable bowel syndrome	10 (17.0)
Episiotomies^a^	7 (17.0)
Forceps deliveries^a^	7 (17.0)
Diabetes	9 (15.5)
Inflammatory bowel disease	3 (5.0)
Anorectal surgery	3 (5.0)
Spinal surgery	3 (5.0)
Rectal Prolapse	2 (3.4)
Anal fissures	2 (3.4)
Scleroderma	2 (3.4)
Radiation therapy (abdomen, pelvis)	2 (3.4)
Rectal/vaginal surgery^a^	1(2.4)
Pelvic or rectal cancer	1(1.7)

Daily incontinence, %	
Solid	17
Liquid/mucous	25

Reason for visit, *n* (%)	
Upper GI complaints	13 (22.0)
Lower GI complaints	36 (62.0)
Liver	5 (8.6)
Anemia	2 (3.4)
FI	2 (3.4)

Duration, *n* (%)	
Less than 5 years, *n* (%)	49 (84.5)
Greater than 5 years, *n* (%)	9 (15.5)

Use of pads, *n* (%)	20 (34)
Use of diapers, *n* (%)	8 (14)

^a^Assessed for women only; percentage represents % of women only

*Percents do not summate to 100% as many patients reported overlapping comorbidities.

**Table 2:** Stool consistency, medication use, severity, and quality of life scores.

Consistency	Medications at the time of visit	Severity score	Quality of life score
Loose/watery —23^a^	None —11 (48%)	29.5	2.26 (1.17–3.92)
	Laxative —6 (26%)		
	Antidiarrheal —7 (30%)		

Formed —17	None —9 (53%)	23.8	23.8 (1.33–3.88)
	Laxative —6 (35%)		
	Anti-diarrheal —2 (12%)		

Hard —6^a^	None —1 (17%)	29.7	29.7 (1.48–3.96)
	Laxative —5 (83%)		
	Anti-diarrheal —1 (17%)		

Alternating —12	None —4 (33%)	29.8	29.8 (1.34–4.07)
	Laxative — 5 (42%)		
	Anti-diarrheal —3 (25%)		

^a^One person took both laxatives and antidiarrheals.

^b^Significant difference between quality of life (QOL) in loose/watery stool consistency versus all other groups combined, *P* = 0.006, and separately (versus formed, *P* = 0.005, versus alternating, *P* = 0.05). No significant difference versus hard stool, *P* = 0.06.

## Appendix

### Background Questionnaire

Data Collection Sheet-1
Fecal incontinence: patient questionnaire
  1. Age: —
  2. Sex: [] M   [] F
  3. Highest education level
    1. [] High school
    2. [] GED
    3. [] College
    4. [] Graduate/higher
  4. Reason for office visit —

(5) Bowel movements:

    — Times/day  — Times/week

(6) Bowel consistency:
  [] Loose or watery
  [] Formed
  [] Hard
  [] Alternating consistency
(7) Past medical history:
  [] Urinary incontinence
  [] Rectal prolapse
  [] Hemorrhoids
  [] Anal fissure
  [] Pelvic or rectal cancer
  [] Diabetes
  [] Anxiety
  [] Scleroderma
  [] Previous Radiation therapy (in abdomen or  pelvis)
  [] Irritable bowel syndrome
  [] Inflammatory bowel disease (Crohn's disease/Ulcerative Colitis)
  [] Previous back injury
  [] Parkinson's disease
  [] Amyloidosis
  [] Depression

(8) Past surgical history:
  [] Anorectal surgery
  [] Spinal surgery
(9) Obstetric history:

Number of pregnancies —

[] Epiostomy  If yes, how many? —
[] Forceps If yes, how many? —
[] Caeserian section If yes, how many? —
[] Normal vaginal delivery If yes, how many? —

(10) Have you ever experienced fecal incontinence (leakage of gas OR soiling with formed or liquid stool OR smearing of undergarments)?
  [] YES   [] NO

Note: if you answered yes to question 10, then answer the following questions, if not you are finished

(11) Age of onset of fecal incontinence —
(12) Duration of fecal incontinence:
  [] Less than 1 year
  [] Between 1–5 years
  [] Greater than 5 years

(13) Use of pad:    [] YES    [] NO
(14) If yes, how often do you use pad?
  — Pads per day
  — Pads per week
  — Pads per month
(15) Use of diaper:  [] YES    [] NO
(16) If yes, how often?
  — Diapers per day
  — Diapers per week
  — Diapers per month
(17) Medications (circle as many as apply, if any):
  [] Laxatives or stool softeners such as: milk of magnesia, lactulose, bisacoydl (dulcolax), castor oil, colace, psyllium (metamucil), senna (senokot), sorbitol
  [] Antidiarrheals such as: pepto-bismol, Imodium (loepramide), lomotil (diphenoxylate)
(18) Have you ever been asked by your doctor about fecal incontinence?
  [] YES   [] NO

Note: if you answered yes to question 18, please answer the following questions.

(19) You were asked by your:
  [] Family doctor/primary care
  [] Gynecologist
  [] Gastroenterologist
  [] Surgeon
  [] Other
(20) Have you ever been evaluated/treated for fecal incontinence?
  [] YES   [] NO
(21) If yes, you have been evaluated/treated by your:
  [] Family doctor/primary care
  [] Gynecologist
  [] Gastroenterologist
  [] Surgeon
  [] Other
